# Formative qualitative assessment of caregiver barriers to childhood immunization in a Peri-urban District in Ghana

**DOI:** 10.1186/s12982-026-01969-0

**Published:** 2026-05-09

**Authors:** Rebecca Chase, Elena Herrera, Lisa Oot, Heather Marlow, TaMiko Condoll, Nessa Ryan, Sofo Ali-Akpajiak, Eliasu Yakubu, Eunice Baiden-Laryea, Paul Konka, Kwame Amponsa-Achiano, Naziru Tanko Mohammed, Shibani Kulkarni

**Affiliations:** 1 JSI Research & Training Institute, Inc., 2733 Crystal Dr 4th Floor, Arlington, VA 22201 USA; 2 Centers for Disease Control and Prevention, 1600 Clifton Rd NE, Atlanta, GA 330333 USA; 3 JSI Research & Training Institute, Inc. Ghana, House No. 1, Odum Street (Norley Road), North Dzorwulu, Accra, Ghana; 4 Saha Consulting & Services Limited, C/O PMB 127 KIA, Accra, Ghana; 5 Centers for Disease Control and Prevention, 24 Fourth Circular Rd, US Embassy, Accra, Ghana; 6 Ghana Health Service, PMB, Dodoo Lane, Osu, Accra, Ghana

**Keywords:** Caregiver-related barriers, Family-based, Male involvement, Vaccination services, Ghana

## Abstract

**Background:**

While access and quality issues in immunization services are well documented, determinants related to social norms and the roles of male and female caregivers remain underexplored in Ghana. This study examined social and behavioral barriers to childhood immunization to inform a family-based intervention to strengthen household engagement in immunization decision-making and improve childhood immunization.

**Methods:**

A qualitative study was conducted in the Kpone Katamanso district of the Greater Accra Region, Ghana, in April 2023. Data was collected in English, Ga, and Twi through 10 key informant interviews with healthcare workers, 10 in-depth interviews with mothers of fully and under-vaccinated children, and eight focus group discussions with mothers (*n*=30) and fathers (*n*=24) of children under two years. Transcripts were coded and analyzed inductively to identify major themes.

**Results:**

Six themes emerged: (1) diverse household immunization decision-making structures; (2) mothers as the primary actors in vaccination; (3) contrasting perceptions of fathers’ versus mothers’ availability; (4) potential to increase fathers’ engagement through improved knowledge and community influencers; (5) differing interpersonal communication experiences with healthcare workers; and (6) health system barriers disproportionately affecting mothers. Social norms and limited male involvement constrained mothers’ time, autonomy, and support, while healthcare barriers, such as staff shortages, indirect costs, and vaccine stockouts, further impeded access.

**Conclusion:**

Findings highlight both demand- and supply-side barriers to childhood immunization. The differential valuation of mothers’ versus fathers’ time emerged as a key structural barrier. Promoting male engagement, strengthening interpersonal communication between caregivers and health workers, and addressing health system barriers may improve immunization uptake and advance family-centered immunization strategies in Ghana.

**Supplementary Information:**

The online version contains supplementary material available at10.1186/s12982-026-01969-0.

## Introduction

Addressing barriers to immunization that may be unique to female caregivers or mothers has the potential to increase immunization coverage across populations, support accessing hard-to-reach communities, and empower women and families to reach their optimal level of health [1, 2]. In the last two decades, childhood deaths from vaccine-preventable illnesses have significantly decreased due to immunizations [1, 3].

Similar trends have been noted in Ghana following the introduction of the Expanded Program on Immunization (EPI) in 1978 [3, 4]. Ghana has maintained high immunization coverage rates, estimated between 90 and 95%, with an estimated 95% for the third dose of diphtheria, tetanus toxoid, and pertussis vaccine (DTP3) in 2023 [5]. However, as of December 2022, there are low-performance districts where children are not receiving the recommended three doses of pentavalent vaccine (Penta3) which includes DTP3, hepatitis B, and *Hemophilus influenzae* type B (Hib) [5–7].

Past Ghana Demographic and Health Surveys from 2014 and 2022–2023 have shown that children under five living in rural areas are more likely to complete the required vaccination schedule put forth by the EPI than children in urban communities [8, 9]. This disparity in immunization coverage may be linked to urban settings facing challenges such as, poor availability and uptake of health services by migrant groups, reduced social cohesion and less structured community organization, and over-burdened health facilities serving a large proportion of the population [10–13].

In many countries across Sub-Saharan Africa and Southeast Asia, females are typically the primary caregivers for children, including managing their healthcare. However, their ability to access immunization services is often hindered by limited decision-making power within the household, insufficient access to and control over economic resources, time constraints, and the burden of heavy workloads both at home and outside the home [14–17]. In many countries, the final decision on vaccinating a child rest with the male as the head of the household [18]. Low decision-making power also negatively affects mobility of women to travel to health facilities or other places outside of the home and can thus negatively impact health-seeking behavior [14–16].

Many women are economically dependent on male counterparts of the household, and this, combined with existing poverty may prevent women from seeking care and immunization services due to lack of monetary resources [19]. Women’s paucity of time and high workload due to multiple responsibilities within and outside the household prevents them from seeking timely vaccinations for their children [19].

Many barriers prevent children from receiving vaccination services, but it is increasingly acknowledged that engaging and involving men/fathers in childhood immunization activities can play a role in potentially reducing barriers faced by female caregivers/mothers [20–22]. Involving fathers may increase awareness of immunization services, promote behavior change in supporting women in decision-making, and/or play an active role in caregivers seeking immunization services [20–22]. For instance, the involvement of fathers in health seeking behaviors, such as accompanying mothers to antenatal care visits, has shown to be associated with greater likelihood of their children being vaccinated [20–22].

While some of these issues are known and studied, other social determinants such as those unique to female caregivers on issues that may affect immunization uptake in Ghana are not well-understood. These issues may highlight the larger cultural context and could directly affect how female caregivers’ access immunization services [23]. This formative assessment was aimed to collaboratively work with Ghana Health Services/EPI to identify female caregiver-related issues that may affect childhood immunization coverage in one peri-urban district in Ghana. One peri-urban district was selected for the assessment to allow for higher methodological rigor. One district allowed for more intensive data collection, monitoring, and process evaluation. This generated stronger evidence about both effectiveness and mechanisms driving success. The formative assessment also intended to identify effective implementation strategies that could be adapted to different district contexts and help inform larger immunization programming in the country.

## Materials and methods

As part of the formative baseline assessment, we conducted a cross-sectional qualitative study in the Kpone Katamanso district of the Greater Accra region of Ghana in April 2023. The study included key informant interviews (KIIs), in-depth interviews (IDIs), and focus group discussion (FGDs) to inform the tailoring of a family-focused intervention aimed at increasing engagement in household immunization decision-making and improving childhood immunization. As part of this assessment, the instruments used were pre-tested and piloted in a randomly selected non-intervention enumeration area to ensure understanding and clarity of content, with sub-districts and subsequent clusters selected in consultation with the district health directorate.

### Setting

While Kpone Katamanso district is predominantly peri-urban in character, it was selected for this study due to its heterogeneous population that includes urban, peri-urban, and rural communities, as well as migrant populations, alongside a high prevalence of un/under-vaccinated children [24].

The district is composed of five sub-districts and 85 communities. Six communities were randomly selected across the five sub-districts for the study (Table 1). Each geographic division in the district had two communities selected, done collaboratively with the district health authorities to ensure equal representation of rural, urban, and peri-urban communities.

**Table 1:** Selected communities and their populations for qualitative study, Kpone Katamanso district, Ghana. Source: District Health Registries, 2023

Name of Community	Sub-district	Settlement geography	Population < 2 years	Number of households
Bawaleshie	Oyibi	Rural	393	711
Okushiribi/Appolonia	Appolonia	Rural	422	729
Halleluyah	Gbetsile	Urban	376	249
School Junction	Zenu	Urban	365	1069
Shangai	Kpone	Peri-urban	429	1416
Switzerland	Zenu	Peri-urban	1153	2003
Total			3138	6177

### Assessment strategies and respondents

The respondent types selected for each evaluation strategy are shown in Table 2.

**Table 2:** Assessment strategies and respondent types for qualitative study Kpone Katamanso district, Ghana

Strategy	Respondent type	*N*
Focus Group Discussion	Male caregivers (*≥* 18 years) of children under the age of two	4 (24 total participants, 5–7 per group)
	Female caregivers (*≥* 18 years) of children under the age of two	4 (30 total participants, 5–9 per group)
Key informant interview	Healthcare workers (s) who provide routine immunization services at Community-Based Health Planning & Services (CHPS) levels	3
	HCWs at sub-district level who supervise routine immunization services	5 (1 per sub-district)
	HCWs at district level who supervise routine immunization services	2
In-depth interview	Mothers of fully vaccinated children under the age of two	5 (rural = 2, urban = 2, peri-urban = 1)
	Mothers of under-vaccinated children under the age of two	5 (rural = 2, urban = 2, peri-urban = 1)

### Data collection

Prior to the start of data collection, ethical approval was received from the Ghana Health Service Ethical Review Committee [ID NO. GHS-ERC 018/05/23] in accordance with the Declaration of Helsinki. The study was conducted in accordance with the Committee's ethical guidelines and regulations of informed consent and ensuring the confidentiality and anonymity of all participants and their information. The U.S. Centers for Disease Control and Prevention’s Global Health Center’s Human Subject Office classified this assessment as a public health activity..

#### Focus Group Discussion (FGDs)

Eight FGDs were conducted with adult (18 years or older) mother (n = 4, 30 total participants) and father (n = 4, 24 total participants) caregivers with children under the age of two; with approximately 5–9 participants in each FGD. Convenience sampling within the six sampled enumeration areas was employed to select participants from their households (Table 1). Separate FGDs were conducted for mothers and fathers to allow participants to feel free to participate actively in the discussions. In this study, the terms ‘father,’ ‘male caregiver,’ and ‘husband’ are used interchangeably to describe adult men who are fathers of children under two and serve as spouses to the mothers in the household. Each FGD was conducted using a semi-structured focus group guide available in English, Ga, and Twi, to minimize interviewer bias and allow participants to share their experiences freely. The guides focused on social norms related to whether families immunize their children; household decision-making regarding childhood immunization; caregiver norms and male involvement in childhood immunization; and barriers and facilitators to childhood vaccination uptake. Data was collected by trained, multilingual interviewers from the local context who were familiar with the cultural norms and languages of the study population accompanied by a trained note taker in a mutually agreed-upon private location and lasted approximately one hour. Interview audio files were recorded via a tape recorder.

#### Key informant interviews (KIIs)

Two district-level health workers responsible for supervising immunization activities in Kpone Katamanso were selected through purposive sampling. Additionally, a random sample of three sub-districts was taken, and from a list of health staff who provide or supervise immunization services at the sub-district and community levels, five were chosen at random for an interview. Random sampling was employed to promote fairness and transparency in participant selection and to minimize bias that might arise if participants were hand-picked by community leaders or health staff. Although qualitative research often relies on purposive sampling, the use of random selection in this context supported balanced participation and strengthened confidence that the views represented were not limited to more vocal or health-motivated individuals. Together, these sampling approaches were designed to be complementary; convenience sampling for FGDs allowed for practical recruitment of caregivers within their own communities to capture a breadth of lived experiences, while random sampling for KIIs ensured that health worker perspectives were systematically obtained across levels of the health system without introducing selection bias. This combination balanced methodological rigor with the practical constraints of field-based qualitative data collection, strengthening the overall representativeness and credibility of the findings. As a result of this sampling, a total of 10 key informant interviews were conducted (KIIs) with healthcare workers at three levels [district EPI (*n* = 2), sub-district health center (*n* = 5), and community health planning and services (CHPS) compounds (*n* = 3)] (Table 2).

The KIIs were conducted using semi-structured interview guides, tailored for respondents at each level of the health system. The purpose of the KIIs was to solicit health worker perspectives on barriers and facilitators to childhood immunization, decision-makers for childhood immunizations, norms, and male involvement in childhood immunization. Key informants were interviewed in an agreed upon private location by trained interviewers in English and interviews lasted between 30 and 45 min. Interview audio files were recorded via a tape recorder.

#### In-depth interviews (IDIs)

IDIs with 10 mothers of children under two were conducted: Five with mothers who had fully vaccinated their children and five with mothers who had not fully vaccinated their children. The purpose of the IDIs was to understand, in detail, from the mother’s perspective their motivations and decisions to fully vaccinate or not fully vaccinate their child(ren). We explored individual experiences with the health system and healthcare providers as well as female and male caregiver issues related to vaccination and barriers and facilitators to vaccination.

The Ghana Health Service (GHS) provided lists of caregivers with fully and partially vaccinated children within the selected enumeration areas (EAs). Based on these records, five EAs with the highest and five with the lowest immunization coverage were purposively selected. Within each of the ten EAs, one caregiver was randomly selected from households with fully immunized children and one from households with partially immunized children, yielding a total of ten caregivers for in-depth interviews. The interview was arranged at a time and private place convenient for the parent. IDIs were conducted using a semi-structured interview guide available in English, Ga, and Twi, and lasted up to 45 min.

Written informed consent was obtained after the nature and possible consequences of the study had been fully explained to the participants. Additionally, note takers took extensive notes during each interview and all interviews were audio recorded. To maintain anonymity, participants were not referred to by name in either the audio recordings or notes.

### Data analysis

Through triangulation across FGDs, KIIs, and IDIs, thematic saturation was reached, with no new major themes emerging by the conclusion of data collection and redundancy of concepts observed across participant groups. Data collectors transcribed the audio recordings for each KII, IDI, and FGD verbatim. Interviews conducted in local languages were translated into English during the transcription process according to best practices in qualitative research [25]. Prior to analysis, all potentially identifying information, such as names, events, or participant occupations were redacted. Data were stored in a secure, password protected folder with access restricted to relevant study staff.

The qualitative analysis software programs NVivo14 and Dedoose were used for coding and analysis, based on differences in software accessibility among the investigation team. Inductive thematic analysis was performed following Castleberry and Nolen’s approach to identify emerging themes related to the experiences and perceptions of each participant group [26]. Each transcript was independently coded by members of the investigation team, and the coding frame and themes were reviewed during regular meetings. Four of the 28 transcripts were double-coded by all investigators to ensure inter-coder reliability and refine the initial codebook. Distinct coding frames were developed for each participant group (mothers, fathers, and health workers) to reflect the unique perspectives and topics discussed within each group. Themes were developed through an iterative process of identifying and organizing codes based on their interrelationship. The investigation team applied a dialogue and consensus approach to resolving intercoder disagreements, where conflicts were discussed until a shared decision was reached [27].

## Results

We identified six themes that captured the different caregiver-related issues with immunization: (1) Diverse childhood immunization decision-making power among caregivers, (2) Mothers as central figure in taking children for vaccination, (3) Differential perceptions of fathers’ vs. mothers’ availability to take children for immunization services, (4) Potential to increase fathers’ engagement in immunization by improving knowledge and awareness through key influencers, (5) Contrasting father vs. mother interpersonal communication (IPC) experiences with healthcare workers, and (6) Health system barriers experienced by mothers. While health system barriers were identified, these results focus primarily on caregiver-related barriers. The thematic structure of the findings, including the relationships between themes, is illustrated in the Appendix (see Appendix, Thematic Organization of Results).

### Diverse childhood immunization decision-making power among caregivers

The findings highlight the diverse dynamics of decision-making within families, differing among communities and households. We found that mothers were typically responsible for making the decision to vaccinate their children, as their role as the main caregiver for the child(ren) gave them this authority.

We heard from fathers that they recognize that mothers often take a lead role in healthcare and vaccination decisions:

*Because they spend more time with the children than we men do. Sometimes before we know what is wrong with the children*,* the mother has already [noticed] it. I believe that is the reason why they take the decision. Sometimes we are at work before they will call us to inform us that this is what is happening. Before they inform us*,* they would have already taken the decision in order to prevent something serious from happening to the child.* (FGD; Father)

However, while this was the consensus across a majority of mothers and fathers interviewed, some caregivers also expressed that the decision is made jointly.

*In my view*,* I don’t think it should be one caretaker. My husband sometimes asks for the child’s CWC card to know what is happening. Even though he is not the one to take the child for vaccination*,* he asks for the carddoes that but I don’t know for the others.* (FGD; Mother)

In some households, mothers emerged as having less authority than other household members, while fathers were the clear figure of authority, including control of vaccination decisions. This power dynamic was described as a cultural custom.

*With regards to the vaccination*,* even if the time is due for vaccination*,* the wife will seek my consent first because I’m the head of the house. Then I will say*,* okay*,* so long as it’s good…because mostly it’s the mothers who take the children to be vaccinated. So*,* once she seeks my concern and I agree to it*,* then she goes to get the child vaccinated. With that*,* it’s mainly on the fathers.* (FGD; Father)

In some instances, religious influence was stated as the reason for male authority:

*From the beginning of creation*,* it is written in the bible that a wife’s head is the Man [husband] and [for the] Man*,* your head is Jesus Christ. So*,* when it comes to such things*,* the wife will seek your consent as the husband*,* and when she does and you know it is good*,* then you have to give her the go ahead to go for the childhood vaccination.* (FGD; Father)

Healthcare workers were also aware of the power dynamic amongst many households:

*Because we know that in Africa especially*,* the man is the head of the house. So if the man says don’t send my child to weighing*,* the woman sits down.* (KII; Sub-municipal Health Staff)

In very few cases mothers went against the fathers opposition and vaccinated their child anyway:

*My husband gets angry if I take our child for vaccination. Because when I take the child for vaccination and we come back home*,* the child will be crying and running a temperature. But*,* I take the child.* (FGD; Mother)

### Mothers as central figure in taking children for vaccination

Findings indicated a notable lack of father’s engagement in taking children to vaccination sessions and highlighted mothers as primarily responsible. This was rooted in social norms around traditional roles for women and men. This is supported by participants’ responses:

*Because I am the mother. I gave birth to him and it is my responsibility to send him any time the date is due.* (IDI; Mother)

Another mother agreed:

*My husband has no idea about vaccination. So*,* if I tell him to take the child for vaccination*,* he will say that he doesn’t know anything about it.* (FGD; Mother)

Fathers similarly expressed that it is not their responsibility to take children to vaccination sessions, and when they do attend, they can be criticized and feel the immunization site is largely a female space that is not particularly welcoming to fathers. A lack of male nurses and low attendance by other fathers were both cited as contributing to the perception that the immunization site was a female space.

*Some will even gossip with friends about you the father for bringing the child for vaccination and making all sorts of allegation such as: has the man divorce the wife or sacked her*, etc. (FGD; Father)

*I don’t recall ever going there to see a male nurse as part of the team doing the CWC session and this is another challenge to be looked at. Because if there are males as part of the nurses*,* again I will feel some sense of belonging to partake in the process.* (FGD; Father)

While mothers are the main caregiver responsible for taking their children to vaccination sessions, the findings indicate that the mothers are looking for further social support from the father for monetary, logistical, and help with vaccination reminders. Two mothers expressed:

*If I have the chance*,* I can also ask my husband to escort me to the hospital. The father can also support with money for transport*, and the other expressed: *The fathers should also assist us in taking the child for weighing. We may be staying far from the hospitals and the roads leading to the facilities are not good. So*,* the fathers can help to drop us at the hospitals with motorbikes if they have one.* (FGD; Mother)

Mothers are also looking for support in the form of reminders and assistance with information retention:

*If there is something going on with the health of the child*,* the father would know directly when he follows me to the hospital and that prevents me from forgetting whatever the nurse told me. So*,* it is better we both go together than just one person going.* (FGD; Mother)

### Differential perceptions and valuation of fathers’ vs. mothers’ availability to take children for immunization services

Mothers and fathers experienced a broad range of time-related barriers to seeking vaccination for their children. Many caregivers indicated that both parents are employed outside of the home, with working hours overlapping with the hours when vaccines are provided:

*By the time I return from work to the place for the vaccination*,* they will say the vaccine is finished.* (FGD; Mother).

In cases where the mother is not employed, responsibilities such as household chores, multiple children in the home, and errands make it challenging to find time to bring their child for vaccination:

*You may have older children and you will have to first attend to them to go to school before you attend to the baby. Washing sometimes*,* you will get tired by the time you finish washing*. (FGD; Mother)

Despite a general perception among both father and mother respondents that mothers have busy schedules, mothers carried a greater expectation than fathers to balance their time to accommodate child vaccination. As noted by a father when asked who is responsible for taking their child for vaccination:

*It’s our wives*,* because we are always busy. Sometimes*,* we even forget that our wives would be going for CWC sessions. We are more focused on the daily bread for the house.* (FGD; Father)

Some fathers stated they believe mothers do not face many burdens or challenges related to the time it takes them to bring their children for vaccination services:

*To me*,* they don’t face much problem because they take the children to the hospital and they vaccinate them and come back home. So to me*,* they don’t face any difficulty*. (FGD; Father).

Some healthcare workers and caregivers noted that fathers are able to “skip the line” at the health facility when they accompany their wives for child vaccination or bring the child themselves. While implemented to encourage male engagement in child vaccination, it may also reflect the greater value that is placed on a father’s time and schedule than a mother’s:

*The men don’t have time unless maybe on that particular day they don’t have anywhere to go… And when they come*,* what we do for them is that… we attend to them first. Then we attend to the rest of the mothers. That is an initiative we are using to get the males also to come and partake in the child welfare activities. We don’t allow the men to wait as the females would wait. Just to encourage more males to patronize it.* (KII; CHPS Health Worker)

### Potential to increase fathers’ engagement in immunization by improving knowledge and awareness through key influencers

Nearly all respondents suggested that various members of the community were capable of encouraging fathers to be more involved in child vaccination. Mothers and healthcare workers most commonly mentioned that the mothers themselves had a strong influence over a father’s engagement. A mother’s close relationship with the father, her intimate knowledge of the child’s health, and her familiarity with the vaccination process were noted as being strong influential factors. In some cases, mothers were considered not just capable of but responsible for educating fathers on the importance of vaccination and encouraging their engagement:

*I am the one who brings the child for weighing*,* so I can tell him [the father] what happens there and that maybe it would be important for him to go to his child’s weighing. So I intentionally give him the weighing card…When he looks through the card then he will be asking about some of the vaccinations that have been given to the child. Then I tell him it helps protect the baby from some of the diseases that affect children during their infant stage.* (IDI; Mother)

Healthcare workers, particularly nurses, were also regarded as key influencers for fathers’ engagement. Their education and expertise lend credibility to the guidance they offer fathers.

*As for we the civilians*,* we don’t really know much about the vaccination.* R*ather*,* it is you the health workers who know much about it. Therefore*,* should there be anything relating to it*,* you are to provide us with the information*. (FGD; Father)

However, given that fathers are not typically present to engage with healthcare workers while their child receives vaccination, there was interest among fathers to receive additional information on immunization, and they suggested specific venues and key people who could engage with them. Churches, community gathering spaces, loudspeaker announcements, and schools were commonly suggested venues for educating fathers.

*I think the education should be more and also be centered on men as well. I think most of the education is targeted to the mothers but I think if the men are involved in it*,* it will surprise you. A man will also take his child to the CWC center to be vaccinated. So*,* the education should be centered on how to get the husbands involved in the vaccination process to help improve it*. (FGD; Father)

### Contrasting father vs. mother interpersonal communication (IPC) experiences with healthcare workers

Mothers and fathers described a broad range of experiences interacting with health workers during vaccination sessions, reflecting the significance of interpersonal communication between caregivers and healthcare workers in both a mother’s and father’s decision whether or not to vaccinate their child. Most of the mothers whose children are fully vaccinated described positive interactions with healthcare workers while attending vaccination sessions. They commonly mentioned that workers provided extensive information about child health and vaccination, addressed the mothers’ questions and concerns patiently, thoroughly, and were friendly.

*Any time I go*,* the people there are always cheerful. Whenever there is a problem and you ask questions*,* they give you the exact answer that you need… they are professionals about their job because whenever you have a problem and you go*,* they are never harsh. They talk to you politely; they relate to you like you are friends with them and I think this is a good thing. (*IDI; Mother)

In some cases, a healthcare worker’s ability to assuage a mother’s fears about possible vaccination side effects contributed to a mother’s vaccine confidence:

*When they come to give the vaccination they tell us*,* ‘Ma’am*,* it’s painful but the vaccination will be of great help to the child.’ I was happy about all that they told me*. (IDI; Mother).

On the other hand, some mothers noted that a healthcare workers’ inability or unwillingness to provide information about their child’s health or vaccination schedule contributed to their negative perception of the vaccination session. For example, a mother of an under-vaccinated child described an experience she had with a healthcare worker who refused to vaccinate her child due to a rash on the child’s body:

*I was expecting them to know the type of rashes my child had*,* but they all said they didn’t know… they just told me to send him to the hospital and should come back when he gets bette*r. *They didn’t give any date*. (IDI; Mother)

Some mothers, particularly those of under-vaccinated children, described being scolded by healthcare workers for missing or delaying their child’s scheduled vaccinations or for not having their child’s weighing book on hand, which discouraged them from attending future vaccination sessions or seeking care at the health facility in general:

*The way they scolded me telling me that ‘even though something was happening to your child you refused to bring him quickly*,* you waited till the time you preferred before bringing him*,*’ that was what I didn’t like*. (IDI; Mother)

### Health system barriers experienced by mothers

Health system barriers such as staff shortages, inaccessibility to health facilities, vaccination costs, and vaccine stock outs were reported to negatively affect vaccine uptake. Mothers experienced these healthcare barriers to immunization, that were not specifically related to the sex of the caregiver but nonetheless impacted them disproportionately because of their status as women in society (Table 3).

**Table 3:** Health system barriers experienced by mothers, qualitative study, Kpone Katamanso district, Ghana

Vaccine stock out^1^	*This is my child. He was one year and three months [old] before he had his nine month injections. So, it is the issue of vaccine shortage that worries me.* (FGD; Mother)
Inaccessibility to health facilities	*Weighing under the tree is not good for us and moreover*,* it is by the roadside. The nurse might have finished attending to your child and you may be doing something so your attention is not on your child. And also*,* a lot of people use the road and then you would have removed all the child’s attire because of the weighing. That is also not good because people can see the child and then spiritually attack the child.* (FDG; Mother)
Distance to the closest facility and poor road conditions	*So*,* some couldn’t get access to the vaccinations. Most of the villages have bad road access. For the nurses and then the doctors to get there is a problem.* (FGD; Father)
Staff shortage	*…In Kpone Katamanso one community health nurse caters to three CHPS due to staff shortage. The work load is huge.* (KII; Health Worker)
Cost^2^	*Our major problem is if you go for weighing*,* you will be asked to pay something. At first*,* they weren’t taking money. Now because of that most parents don’t send their children for vaccination as they don’t have [money] and feel shy to go.* (IDI, Mother)

^1^ Referred to as a vaccine shortage, meaning when a specific vaccine is completely absent and unavailable at a given facility or vaccination session

^2^ Vaccination services are free through the Ghana Health Service, however HCWs often ask mothers for funds to cover the cost of tables and chairs for the sessions, and small incentives for themselves

## Discussion

This assessment of caregivers and health workers identified key social and behavioral factors influencing household immunization decision-making and practices in Kpone Katamanso, Ghana. Similar to studies conducted in other sub-Saharan African settings [18–20], norms around caregiving and social roles for males and females strongly shaped vaccination behaviors. However, unlike research from Kenya and Uganda, where fathers or senior women commonly dominate household health decisions [18, 19, 22], mothers in this peri-urban Ghanaian district reported greater participation in immunization decision-making. This likely reflects local socioeconomic dynamics, as women in Kpone Katamanso frequently engage in formal employment and exhibit higher levels of autonomy compared to women in more rural or traditional communities [24].

Despite this relative empowerment, mothers continued to bear primary responsibility for vaccination logistics, tracking immunization schedules, transporting children to clinics, and managing competing household and occupational demands. This finding aligns with prior research from Nigeria, where child health is viewed as a mother’s exclusive domain, even in households requiring a father’s approval for healthcare decisions [28–30]. Consistent with studies from Gabon and Uganda [20, 31], mothers in our study reported time constraints, long waiting periods, and competing domestic priorities as barriers to vaccination adherence. Interestingly, participants perceived fathers’ time as more valuable than mothers’, even when both parents were employed. This reflects persistent social hierarchies and unequal valuation of men’s versus women’s labor, a pattern similarly noted in sex and health access research from Uganda and Kenya [20, 22].

Health system challenges further compounded these barriers experienced differently by male and female caregivers. While this study focused primarily on caregiver-related barriers, the health system challenges identified, including staff shortages, vaccine stockouts, and inadequate interpersonal communication, emerged as critical contextual factors that interact with and exacerbate household-level barriers; particularly for mothers who bear the primary responsibility for navigating these system-level constraints. Although immunization services in Ghana are officially free, mothers described indirect costs including transportation, requests from HCWs for payment to cover facility expenses such as chair and table rental fees, as well as service inefficiencies such as vaccine stockouts and long queues. These health system factors amplify the imbalance of household dynamics where mothers continue to depend on the fathers for financial resources for immunization. Long waiting times, erratic supply of vaccines, missed opportunity for income generation, and long distances to outreach points not only decrease mothers’ financial autonomy but also contributes to time paucity and increased household workload, which forms a distinct burden on mothers. These barriers mirror findings from Gabon, Haiti, Turkey, and Uganda, where structural and economic constraints limited women’s ability to access vaccination services for their children [31–34]. Such systemic issues not only reinforce difference related to sex but also undermine trust in the health system, highlighting the importance of addressing both supply- and demand-side determinants simultaneously.

Low male engagement emerged as a key behavioral gap. Fathers generally supported vaccination in principle but lacked practical knowledge about vaccines and the immunization process, echoing prior studies linking low parental knowledge, particularly among male caregivers, to incomplete immunization coverage [35, 36]. Similar to evidence from Kenya and Nigeria [21, 30], sociocultural norms discouraged fathers from participating in child health visits, as such involvement was often perceived as socially inappropriate or subject to gossip. However, fathers in this study expressed openness to engagement if the environment were more welcoming and inclusive. This reinforces Goodman et al.’s argument that mainstreaming sex specific considerations in immunization policy, through the inclusion of both male and female caregiver perspectives, can improve service design and reduce inequities [37].

All together, these findings suggest that social norms for male and female caregivers in peri-urban Ghana are shifting toward more collaborative decision-making yet remain constrained by traditional expectations and systemic barriers. Interventions that promote targeted health education for males and females, target fathers with tailored vaccine information, and strengthen interpersonal communication between health workers and caregivers could help normalize shared parental responsibility. As seen in other African contexts, engaging trusted community figures such as religious and traditional leaders may be an effective strategy for improving male participation and, ultimately, child immunization coverage [20, 35, 36].

These assessment findings, can help Ghana EPI inform comprehensive policies that address both demand and supply-side barriers to childhood immunization. National immunization policy could consider the integration of father-specific educational content into existing community health education programs. Programmatically, cadre of health care and community health workers could be trained in recognizing the need for family involvement and engagement. Training modules on how to effectively communicate with fathers and mothers on the importance of childhood immunization, explore ways in which fathers could support immunization, and offer guidance and support to fathers could be developed. This content could be delivered through churches, schools, and community platforms by trained religious leaders, community leaders, and healthcare workers, as research in sub-Saharan Africa has demonstrated that low parental knowledge, particularly among male caregivers, contributes significantly to under-vaccination [35, 36]. EPI guidelines could standardized interpersonal communication training for all immunization staff to ensure respectful, non-judgmental interactions with caregivers. Positive provider-caregiver relationships have been shown to enhance vaccine confidence and sustained health-seeking behavior [17, 20]. Capacity building efforts and community engagement strategy for National Community Health Planning and Services structures could incorporate male involvement when conducting regular outreach for maternal and child health issues. Policy reforms at the larger health system level to eliminate informal payment practices at vaccination sites, strengthen vaccine supply chain management, address staff shortages, and improve facility accessibility may also eventually support mothers to complete immunizations for their children. These structural barriers disproportionately affect mothers and undermine the principle of free immunization services [7, 31–34]. Finally, national immunization policy could consider formally adopting family-centered approaches that recognize diverse household decision-making patterns. These approaches should promote collaborative immunization decisions between mothers and fathers, moving beyond traditional maternal-child health frameworks to engage the entire family unit in childhood health [21, 29, 37]. Further policy implications will emerge after interventions are implemented and their effectiveness is systematically analyzed and evaluated.

## Study limitations

This study has a few limitations that should be noted. Interviews were conducted among a small sample in one district of Ghana; therefore, results may not be fully representative of all Ghanaian caregiver experiences. The cross-sectional nature of the study also limits the ability to draw causal inferences about the relationship between identified barriers and immunization outcomes. Given the qualitative nature of this study, participants were asked to self-report their experiences, attitudes, and beliefs related to childhood immunization, which may have introduced social desirability bias. Additionally, as data were collected in multiple languages (English, Ga, and Twi) and involved local data collectors, the potential for observer bias and inconsistencies in translation and interpretation cannot be fully excluded, despite efforts to ensure rigor through structured training and review processes. Finally, fathers were underrepresented in the sample relative to mothers, with 24 fathers compared to 30 mothers participating in focus group discussions. This may have limited thematic saturation for male perspectives and constrained the depth of insight into paternal attitudes and decision-making roles in childhood immunization.

## Conclusion

Findings from this formative qualitative assessment will help to inform and tailor intervention strategies for male engagement for immunization. Researchers from this formative assessment will collaborate with local stakeholders to co-design and implement these interventions, followed by further evaluation to determine their effectiveness. Given the importance of ensuring all children receive routine immunizations and caregivers receive appropriately targeted communications, the results of this study are important. Through triangulated data from mothers, fathers, and healthcare workers, this assessment provides in-depth examination of both maternal and paternal perspectives on immunization barriers in a peri-urban Ghanaian context. It reveals the nuanced decision-making dynamics that vary across households within the same district. Additionally, it identifies the differential valuation of mothers versus fathers’ time as a previously underexplored barrier to immunization uptake.

Based on our findings, interventions should address barriers specific to female and male caregivers that could affect immunization uptake at the household, community, and health system levels. Specifically, given that fathers expressed discomfort attending vaccination sessions due to the predominantly female environment and lack of male healthcare workers (Theme 5), interventions should work to make immunization sites more welcoming to fathers while enhancing their support to mothers for childhood immunization. Since nearly all fathers reported low knowledge about vaccines and the vaccination process (Theme 4), interventions should prioritize improving fathers’ knowledge and awareness of vaccine services through influential community members such as religious leaders, healthcare workers, and community leaders. As mothers identified the need for assistance with vaccination reminders, transportation, and information retention (Theme 2), interventions should increase fathers’ social support in these practical areas. Finally, since decision-making patterns varied across households, with some mothers making decisions independently while others required male approval (Theme 1), interventions should promote joint decision-making between fathers and mothers to ensure collaborative approaches to childhood immunization.

Future research should focus on several key areas to build upon these findings. First, longitudinal studies are needed to measure the impact of family-centered interventions on immunization demand, uptake, and coverage rates in peri-urban settings. Second, implementation science research should evaluate the feasibility, acceptability, and scalability of male engagement strategies across diverse district contexts in Ghana, including comparative analyses across urban, peri-urban, and rural settings. Finally, cost-effectiveness analyses of interventions targeting male engagement versus traditional maternal-focused approaches should inform resource allocation decisions for the Ghana EPI program. While the themes identified in this study are common across populations, this data cannot be extrapolated specifically to other populations without similar contextual assessments [23, 38]. Overall, this formative assessment highlights the importance of understanding context-specific caregiver-related barriers and informs the interventions that can be implemented to address them.

## Supplementary Information

Supplementary Material 1

## Data Availability

The datasets used and/or analysed during the current study are available from the corresponding author on reasonable request.
