# Supplementary material for: Formative qualitative assessment of caregiver barriers to childhood immunization in a Peri-urban District in Ghana
Source: Discov Public Health. 2026 May 9;23(1):689. doi: 10.1186/s12982-026-01969-0 (PMC13157455; doi:10.1186/s12982-026-01969-0)
Supplement: Supplementary file 1 — Supplementary Material 1 [file 12982_2026_1969_MOESM1_ESM.docx]

**Formative Qualitative Assessment of Caregiver Barriers to Childhood Immunization in a Peri-urban District in Ghana**

*Thematic Organization of Results*

### **1.**

### Diverse Childhood Immunization Decision-Making Power Among Caregivers

**2.**

Mothers as Central Figure in Taking Children for Vaccination

**3.**

Differential Perceptions and Valuation of Fathers’ vs. Mothers’ Availability for Immunization Services

**4.**

Potential to Increase Fathers' Engagement by Improving Knowledge and Awareness through Key Influencers

**5.**

Contrasting Communication Experiences Father vs. Mother Interpersonal Communication with Healthcare Workers

**6.**

Health System Barriers

**2**

**Mothers as central figure in taking children for vaccination**

**3**

**Differential perceptions a**
